# The effect of cryotherapy on fibromyalgia: a randomised clinical trial carried out in a cryosauna cabin

**DOI:** 10.1007/s00296-018-4176-0

**Published:** 2018-10-23

**Authors:** Javier Rivera, María José Tercero, Javier Salas Salas, Julio Hernández Gimeno, Javier Sánchez Alejo

**Affiliations:** 1 Reumatología, Instituto Provincial de Rehabilitación, Francisco Silvela 40, 28028 Madrid, Spain; 2 Tecnología e Innovación Médico-Estética, S.L., Madrid, Spain; 3 Policlínica Meprysa, Madrid, Spain

**Keywords:** Fibromyalgia, Clinical trial, Cold therapy

## Abstract

Evidence of symptomatic treatment for fibromyalgia (FM) is very low. Whole body cryotherapy (WBC) modulates different neurotransmitters, which might have a role in pain alleviation and could exert an effect on FM. Our aim was to evaluate the efficacy of WBC for the control of pain and impact of disease in FM. For this we run an open, randomized, crossover trial of Cryosense TCT™ cabin vs rest. Patients with FM according to ACR criteria were recruited consecutively from general practices. Trial endpoints were change (∆) in pain after 2 and 4 weeks, measured by a visual analogue scale (VAS), ∆ burden of disease, evaluated by the Fibromyalgia Impact Questionnaire (FIQ), and severity of FM, measured by the Combined Index of Severity of Fibromyalgia (ICAF). Within group differences, sequence and period effects were tested with Student’s *t* or Mann–Whitney *U* tests. Multiple linear regression models were used to adjust effect by baseline differences between groups. Sixty patients were included in the trial. A period effect was noted, with residual effect of WBC; therefore, only results from the first sequence were analysed. ∆VAS pain, ∆FIQ and ∆ICAF scores were significantly larger in the WBC group after the first period (3.0 vs 0.3 in ∆VAS pain; 32.1 vs 0.4 in ∆FIQ; 13.7 vs 0.07 in ∆ICAF; all *p* < 0.001), and were confirmed after adjustment. In conclusion, WBC with a Cryosense TCT cabin may be a useful adjuvant therapy for FM; further studies on long-term effect and compared to other physical therapies are warranted.

*Trial registration* NCT03425903.

**Electronic supplementary material:**

The online version of this article (10.1007/s00296-018-4176-0) contains supplementary material, which is available to authorized users.

## Introduction

Fibromyalgia (FM) is a syndrome characterised by chronic generalized musculoskeletal pain, hyperalgesia, and allodynia. Musculoskeletal pain is usually accompanied by fatigue, sleep problems, anxiety and depression, paresthesiae, joint stiffness, headache, subjective sensation of swelling, concentration difficulties, and memory impairment, among other unexplained symptoms [1, 2]. FM has an elusive aetiopathogenesis; very likely, genetic and environmental factors, not exclusive to FM [3], determine a persistent dysfunction in systems modulating pain, such as central nociceptive hyperexcitability—or amplification—and decreased inhibitory response [4–6]. An individual may develop a picture of FM from different pathways [7], including trauma, sleep disorders, or an altered neuroendocrine stress response [5, 6, 8], making difficult to untangle whether FM leads to the constellation of symptoms or vice versa.

The natural course of FM is chronic, and fluctuating, becoming disabling in some individuals, with a devastating effect on people’s lives, affecting their ability to work and engage in everyday activities, as well as their relationships with family and friends. The limitation in the performance of activities of daily living, together with its high prevalence—it affects 2% or more of the population [1, 9]—, makes FM a first magnitude health problem that imposes large economic burden on society [6, 10].

Although there are several drugs and other non-pharmacological measures available for the management of fibromyalgia, at present there is no a definitive cure. Some drugs act on pathogenic mechanisms of pain processing while most non-pharmacological measures, as well as other drugs, provide mainly a symptomatic relieve [11]. Experts recommend to focus on patient education and non-pharmacological alternatives as initial management, followed by tailored psychological therapies, analgesic or sleep modulating remedies, or multimodal rehabilitation in the last step [12]. Due to the limited efficacy of the therapeutic options, patients usually seek help in alternative therapies, like acupuncture, biofeedback, chiropractic, hypnosis, transcranial direct current stimulation, etc. [5, 12, 13].

Cryotherapy refers to the application of cold as a therapeutic agent for pain relief, a remedy widely used in sports related trauma, based on its capacity to decrease the inflammatory reaction, including oedema [14]. Cold packs have been widespread accepted for the treatment of musculoskeletal pain despite moderate evidence supporting effectiveness [15]. Whole-body cryotherapy (WBC) involves exposure to extremely cold dry air in an environmentally controlled chamber or cabin for short periods of time (between 2 and 5 min) [16]. WBC reduces inflammation, and produces analgesia through neuroreflexive processes by decreasing skin temperature, for which it has been tested as a recovery technique after exercise [16], and in rheumatic and inflammatory diseases such as rheumatoid arthritis [17], and ankylosing spondylitis [18].

Chryotherapy induces many physiological reactions in the organism with an increase in white blood cells, antiinflammatory cytokines, ACTH, beta-endorphins, cortisol and catecholamines. Another possible mechanisms of action of cryotherapy have been postulated and include immunostimulation due to noradrenalin response to cold, reduction of pain through the alteration of nerve conduction, an increase in the level of plasma total antioxidant status and improving immune function [19].

Muscle activity and inflammation produce oxidants in the intercellular space with the consequences of membranes damage as well as more inflammation in a vicious cycle. Through a reduction of inflammation, cold has been suggested to reduce oxidants production [19].

The reduction of pain in FM produces a chain reaction, triggering improvement in mood, leading in turn to better pain adjustment and facilitation of physical and mental activities, all of which justify testing therapies that may reduce pain without harming the patient. Exercise produces the largest effect on pain in FM [20], but post-exertional pain and fatigue is a salient feature in FM. For this reason, low intensity exercise is used as mainstream therapy [21], and in this context, adjuvant therapy for muscular soreness, such as cold therapy, may be rationalised.

The scientific evidence about efficacy of cryotherapy in fibromyalgia patients is limited to an observational study that showed promising results [22]. The purpose of the present study is thus to provide additional evidence on the effects of cryotherapy in the clinical manifestations of FM patients.

## Methods

This was a randomised, open, crossover trial of 3 weeks duration (7 weeks in both periods included) to test the efficacy of WBC as adjuvant therapy for the control of pain and impact of disease in patients with FM. The study protocol and materials were approved by the Clinical Research Ethics Committee of the Hospital Puerta de Hierro of Madrid (TIME-CRY-2015-01, V04 JULIO 2016-Meeting 2016-07-11).

Eligible patients were recruited consecutively from participating general practices. Selection criteria included: age between 25 and 80 years old; diagnosis of FM according to ACR criteria [2]; more than 1 year from diagnosis; lack of response or partial response to previous treatment; in case of women, commitment not to get pregnant during the study. Participants were excluded if they had cardiovascular or psychiatric comorbidity, cold intolerance, changes in pharmacological or non-pharmacological treatment during the study—including treatment changes at baseline—or a body temperature over 37.5 °C.

After inclusion, subjects were randomly assigned to a sequence starting by WBC or control using a randomization scheme generated through the http://www.randomization.com website [23].

**Fig. 1:**
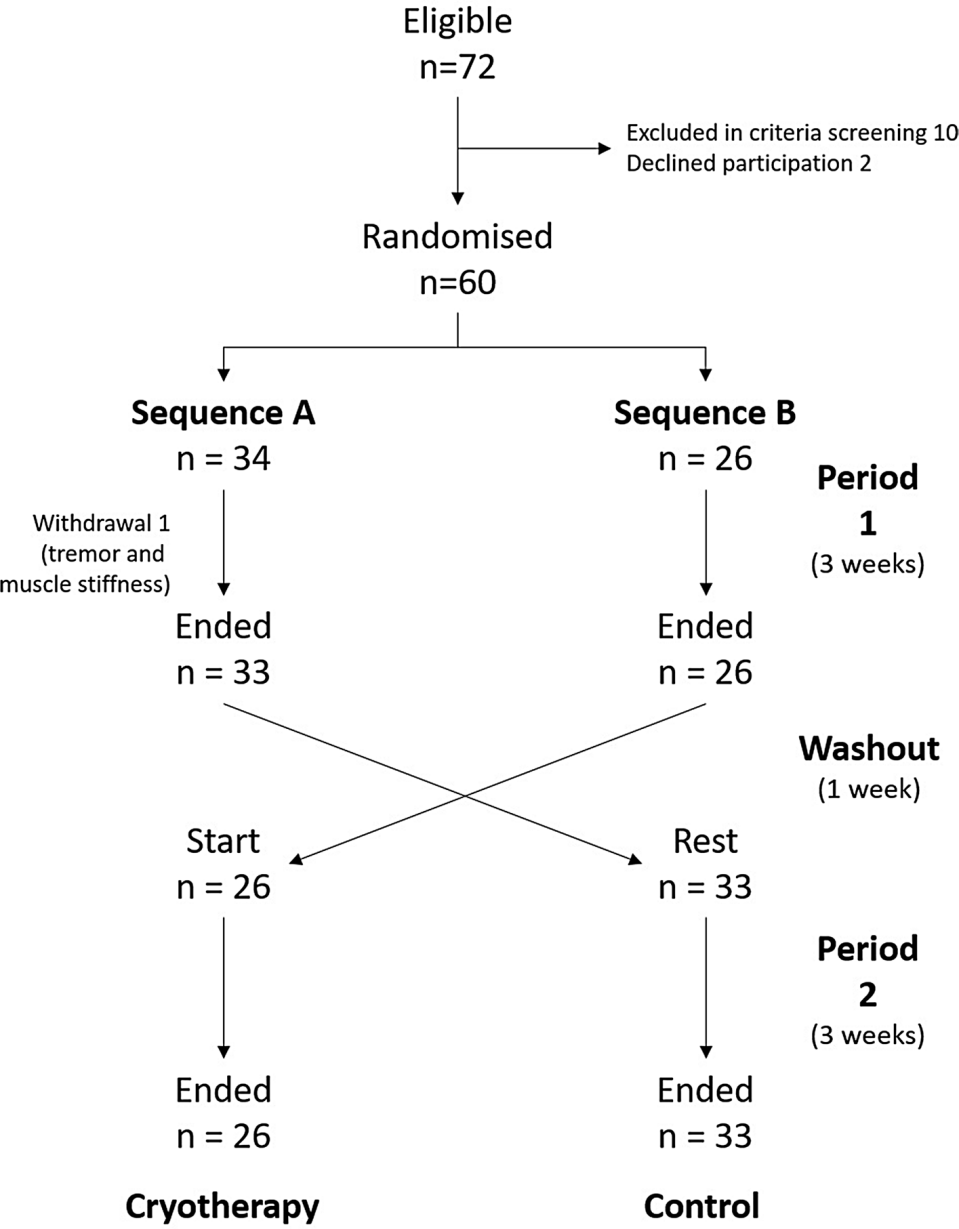
Flow-chart of participants through the trial

Patients on WBC group were treated on alternate days during 3 weeks. At each session, patients were introduced in Cryosense TCT™ cabins during 3 min, where temperatures reached − 196 °C—the evaporation point of liquid nitrogen—. After ten sessions, patients underwent a 1-week washout period to eliminate any possible residual effect of the previous application of WBC. Subsequently, the groups were inverted; those initially treated with WBC became controls and vice versa (Fig. 1). In addition, patients maintained current treatment (pain-killers on a regular basis) without modification during the duration of the study.

**Fig. 2:**
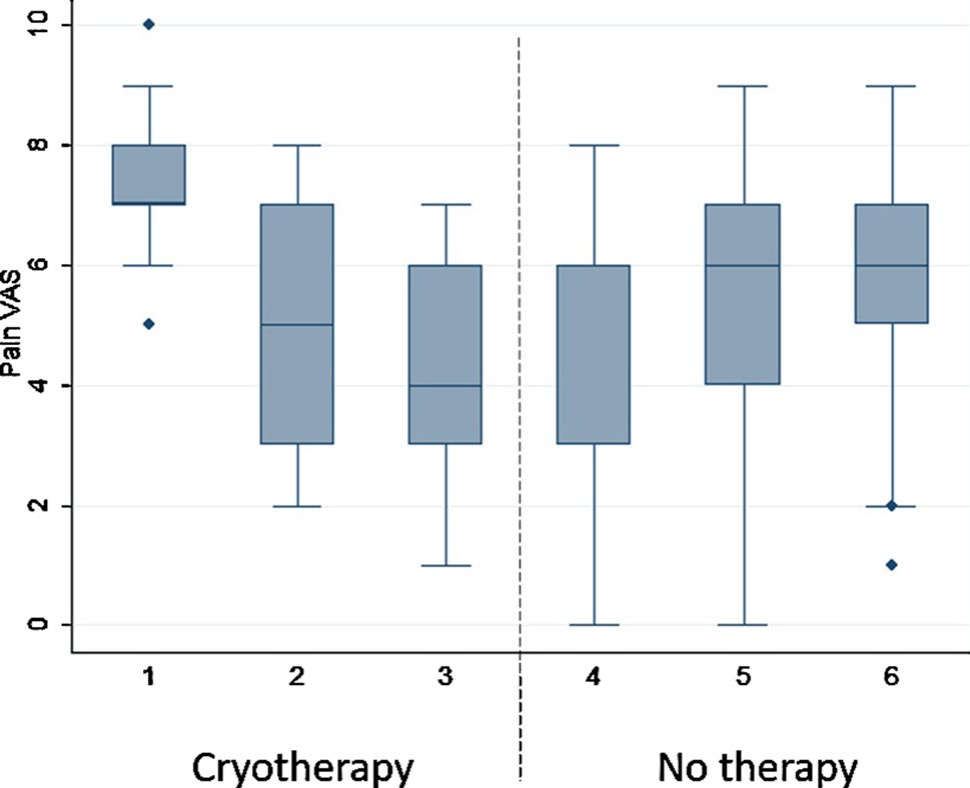
Evolution of the pain VAS along the study visits (7 weeks) in the group initially assigned to cryotherapy. Visit 4 corresponds to the end of washout period and visits 5 and 6 to control period (no cryotherapy). Visit 6 occurs 4 weeks after the last session of cryotherapy. Diamonds (*τ*) represent outliers. In visit 4, median overlapped with lower quartile (bottom part of the box)

Trial main endpoints were changes in pain, assessed by a 10 cm visual analogue scale (VAS), and in impact of disease, assessed by the Fibromyalgia Impact Questionnaire (FIQ) [24]. As secondary endpoint, changes in the severity of the disease were tested, assessed by the Combined Index of Severity of Fibromyalgia (ICAF) [25], and on the SF-36. The FIQ is a self-administered questionnaire that tests the ability to perform large muscle tasks, difficulty with work, pain, fatigue, morning tiredness, stiffness, anxiety and depression; it contains ten items with a range of scores from 0 to 100, with higher score indicating greater impact.

On the other hand, ICAF is a tool for assessing the severity of FM based on its most prevalent clinical manifestations, resulting in a total score of the severity where higher scores represent greater importance of the condition and its consequences in the patient’s life. The ICAF questionnaire also provides information on emotional, physical, and of coping (active and passive) aspects of the patient. The emotional factor emphasizes the role of emotional aspects such as anxiety and depression; the physical factor assesses pain, fatigue, sleep quality and functional ability; active coping includes positive coping strategies, and passive coping identifies a group of particularly severe patients. The ICAF contains 59 items and its score ranges from 0 to 84, with higher values indicating greater severity [26].

All patients were assessed after 22 and 50 days from period start—visits 3 and 6, corresponding to the evaluation of the first and second periods, respectively—(see Table 1 at supplementary material for an outline of study procedures and assessments).

**Table 1:** Baseline characteristics by treatment group

Visit 1 (baseline)	Cryotherapy (*n* = 34)	Control (*n* = 26)	*p* value
VAS pain	7.3 ± 1.3	6.6 ± 1.5	0.052
FIQ	73.8 ± 13.4	64.1 ± 15.7	0.012
ICAF total	49.7 ± 9.6	44.6 ± 8.9	0.043
ICAF (physical)	52.9 ± 7.9	48.0 ± 10.7	0.045
ICAF (emotional)	49.2 ± 9.0	44.1 ± 9.9	0.041
ICAF (active copying)	53.8 ± 10.2	54.9 ± 6.9	0.658
ICAF (passive copying)	52.2 ± 10.6	50.8 ± 9.6	0.613
SF-36 physical component	35.2 ± 6.0	28.9 ± 5.7	< 0.001
SF-36 mental component	37.4 ± 5.4	38.8 ± 12.0	0.596
SF-36 (physical functioning)	38.7 ± 23.5	40.0 ± 19.6	0.827
SF-36 (role physical)	27.0 ± 28.0	8.6 ± 18.6	0.001
SF-36 (bodily pain)	37.2 ± 20.9	24.4 ± 17.3	0.014
SF-36 (general health)	52.9 ± 20.0	37.5 ± 20.0	0.004
SF-36 (vitality)	40.1 ± 11.4	26.7 ± 14.9	< 0.001
SF-36 (social functioning)	53.7 ± 9.5	42.8 ± 27.0	0.278
SF-36 (role emotional)	21.6 ± 33.7	56.4 ± 46.0	0.002
SF-36 (mental health)	60.6 ± 11.1	52.9 ± 18.6	0.070
Visit 4 (baseline after washout)			
VAS pain	5.0 ± 2.2	6.5 ± 2.5	0.013

*VAS* visual analogue scale, *SF-36* short-form questionnaire, *ICAF* combined index of severity of fibromyalgia, *FIQ* fibromyalgia impact questionnaire

Secondary endpoints were 50% reduction of pain at days 10 and 22, and changes in quality of life (SF-36) [27].

### Statistical analysis

A sample size of 60 participants was deemed sufficient to detect a significant treatment group difference on pain VAS, accounting for a dropout rate of 20% (power 80% and alpha level of 0.05).

The sample was described by summary statistics (mean and standard deviation, frequencies and percentages). Differences between groups at baseline (visit 1 and visit 4, or baseline after washout) were tested with Student’s *t* or Mann–Whitney *U* tests, depending on the distribution of continuous variables, and Chi square for qualitative variables. Normality was tested with the Kolmogorov–Smirnov test.

Within-group differences in outcome measures by time were assessed using repeated measures ANOVA.

Sequence and period effects were evaluated. The comparison of response in terms of ∆VAS pain and ∆FIQ, and of the secondary outcomes, between treatment groups was carried out using Student’s *t* or Mann–Whitney *U* tests, depending on the distribution of the respective variables, with an intention to treat approach.

Finally, and to take account possible differences in baseline between both study groups, multiple linear regression was used. Models were constructed using the outcome measures as dependent variables, and treatment group, controlling for variables with significant baseline differences (complete model), as independent variables. Backward stepwise regression was used for modelling variables selection with successive elimination of those without confounding effect. Comparison of models was performed by information measures, Akaike information criteria (AIC), and Bayesian information criteria (BIC). The final models were the most parsimonious and with the lowest values on information criteria.

## Results

Eligibility criteria were fulfilled by 72 patients of whom 12 were excluded for not fulfilling all criteria or declining participation, leaving 60 patients, whose randomisation resulted in two groups of 34 and 26 patients, assigned to WBC and control, respectively (See Fig. 1). One patient received two sessions and left the study. His baseline data were carried forward to the rest of the follow-up, regardless the period.

The distribution of the VAS, FIQ, ICAF and SF-36 total scores was normal. Comparability between groups was tested on visits 1 and 4, corresponding to the beginning of both periods. As of visit 1, groups differed in FIQ score, ICAF (total, physical, and emotional subscales), SF36 dimensions (physical function, body pain, general health, vitality, emotional role) and standardized total score of the physical component, with consistently higher values for the WBC group, which could indicate a worse baseline clinical situation for this group, whereas no differences were found in pain score. In addition, there were no differences between groups in the total number of medications consumed by the patients at baseline (all patients consumed at least one pain-killer on a regular basis), as well as in the number of drugs for treating other comorbidities.

VAS pain at visit 4, after washout, showed a significant difference between groups with lower values in the WBC group compared to control (5.0 vs 6.5; *p* = 0.013), which reflects a residual effect of therapy (see Table 1; Fig. 1).

The difference or change of score between baseline and the evaluation visit was used as the outcome variable; that is, the differences between visit 1 and visit 3, for the first period, and between 4 and 6 for the second one. There was no sequence effect. However, a period effect was observed with significant differences between the responses of the first and second period in the main outcome measures: VAS pain (*p* = 0.015) and total FIQ (*p* value = 0.003). That is, individuals did not return to baseline situation after the first treatment. Therefore, the second period after cross-over could not be included in the analysis.

During the first study period, intra-group differences over time were observed in three outcome measures (∆pain VAS; ∆FIQ and ∆ICAF) in the intervention group, but not in the control group (Table 2) (Fig. 2).

**Table 2:** Within-group differences by time

	Group	VAS pain^a^	*p* value	FIQ total^a^	*p* value	ICAF^a^	*p* value
V1	Intervention	7.3 ± 1.3	< 0.0001	73.8 ± 13.4	< 0.0001	49.7 ± 9.6	< 0.0001
V2		5.1 ± 2.1		48.9 ± 18.7		–	
V3		4.4 ± 1.9		41.6 ± 20.4		36.0 ± 10.1	
V1	Control	6.6 ± 1.5	0.629	64.1 ± 15.7	0.792	44.6 ± 8.9	0.939
V2		6.3 ± 2.1		–		–	
V3		6.3 ± 2.3		63.6 ± 16.4		44.7 ± 8.5	

*V* visit, *VAS* visual analogue scale, *FIQ* fibromyalgia impact questionnaire, *ICAF* combined index of severity of fibromyalgia

^a^Results presented as mean ± standard deviation

The results of the first period showed a significant effect on VAS pain and FIQ score of WBC (∆ VAS pain 3.0 vs 0.3, and ∆ FIQ 32.1 vs 0.4; both *p* < 0.0001). Similarly, the change in ICAF score, both total (∆ ICAF total 13.6 vs − 0.07) and by domains (emotional factor, physical factor, active coping and passive coping), was significantly greater in the treated group than in the non-treated. Finally, no significant changes were observed in SF-36 dimensions between groups, except in the physical function and in the emotional role, which presented larger changes in the WBC group than in the control group (Table 3).

**Table 3:** Treatment effect on trial endpoints and secondary outcomes: first period (V1–V3)

	Total (*n* = 60)	Cryotherapy (*n* = 34)	Control (*n* = 26)	*p* value
VAS pain	1.8 ± 2.4	3.0 ± 2.3	0.3 ± 1.6	< 0.0001
FIQ	18.4 ± 21.9	32.1 ± 18.9	0.4 ± 8.2	< 0.0001
ICAF total	7.7 ± 10.0	13.6 ± 8.9	− 0.07 ± 4.6	< 0.0001
ICAF (physical)	11.4 ± 13.7	19.5 ± 12.3	0.8 ± 6.1	< 0.0001
ICAF (emotional)	5.5 ± 8.3	9.5 ± 8.1	0.3 ± 5.1	< 0.0001
ICAF (active copying)	− 1.6 ± 7.3	− 4.0 ± 8.0	1.4 ± 5.1	0.002
ICAF (passive copying)	3.0 ± 9.7	5.4 ± 11.0	− 1.3 ± 6.8	0.021
SF-36 physic component	− 1.1 ± 6.8	− 1.7 ± 7.9	− 0.4 ± 4.9	0.426
SF-36 mental component	− 3.8 ± 9.1	− 5.4 ± 9.6	− 1.6 ± 7.9	0.102
SF-36 (physical functioning)	− 11.5 ± 17.7	− 19.6 ± 16.8	− 0.8 ± 12.4	< 0.0001
SF-36 (role physical)	− 7.2 ± 36.8	− 8.3 ± 43.3	− 5.8 ± 26.7	0.819
SF-36 (bodily pain)	− 6.6 ± 23.0	− 9.0 ± 29.4	− 3.6 ± 9.2	0.327
SF-36 (general health)	6.6 ± 29.5	9.8 ± 37.2	2.4 ± 14.2	0.291
SF-36 (vitality)	− 5.7 ± 14.4	0.8.4 ± 17.4	− 2.1 ± 8.3	0.071
SF-36 (social functioning)	− 7.3 ± 22.9	− 8.8 ± 24.5	− 5.3 ± 21.0	0.558
SF-36 (role emotional)	− 23.3 ± 45.6	− 40.2 ± 41.7	− 1.3 ± 41.6	0.001
SF-36 (mental health)	− 1.5 ± 16.7	0.0 ± 19.7	− 3.5 ± 11.8	0.393

Results are expressed as change or difference between the scores of visits 1 and 3 (first period)

*VAS* visual analogue scale, *SF-36* short-form questionnaire, *ICAF* combined index of severity of fibromyalgia, *FIQ* fibromyalgia impact questionnaire

The objective of the multivariate regression models was to control the possible confounding effect of those variables that showed baseline differences between study groups due to unbalanced randomization. Therefore, in the first model (saturated) all those variables with significant basal differences were introduced (Table 1). However, a successive elimination of those variables without confounding effect on the association between outcome measure and study group was carried out in order to obtain a more parsimonious model that would better fit a limited sample size. The final models are those shown in Table 4. The obtained results confirmed the significance difference in VAS pain (*β* = 2.56), FIQ score (*β* = 29.7), and ICAF (*β* = 12.8), independently of baseline values. Finally, the models explained 45.5% of the variance of ∆VAS pain, 54.3% of the variance of ∆FIQ score, and 47.6% of the variance of ∆ICAF (Table 4).

**Table 4:** Adjusted treatment effects: linear regression

Variables	VAS pain (∆V1 − V3) *β* (*p* value)	FIQ total (∆V1 − V3) *β* (*p* value)	ICAF total (∆V1 − V3) *β* (*p* value)
Treatment group Control Cryotherapy	1 **2.56** (< 0.0001)	1 **29.7** (< 0.0001)	1 **12.8** (< 0.0001)
VAS pain	0.89 (0.001)		1.16 (0.096)
FIQ		0.21 (0.132)	
ICAF (physical)	− 0.10 (0.006)		
ICAF (emotional)			
SF-36 (role physical)			
SF-36 (bodily pain)			
SF-36 (general health)			
SF-36 (vitality)			
SF-36 (role emotional)			
Constant	− 0.60 (0.665)	− 13.0 (0.166)	− 7.70 (0.109)

Each column contains the multivariate model. The effect of treatment is the beta coefficient of the treatment group (in bold). *R*^2^ = 45.5% for VAS pain; 54.3% for FIQ score; and 47.6% for ICAF total

*VAS* visual analogue scale, *SF-36* short-form questionnaire, *ICAF* combined index of severity of fibromyalgia, *FIQ* fibromyalgia impact questionnaire

### Adverse events

Five patients referred adverse events during cryotherapy, including: heartbeat feeling in whole body (1), palpitations (1), sleep difficulties (2), bowel sounds and bloating (1), muscle stiffness (1), tremor (1), headache (1). All of them were mild and appeared during the first sessions, waning afterwards. In one patient, muscle stiffness and tremor obliged the discontinuation of therapy after two sessions. Adverse events were recorded prior and after each session, therefore, there is no information on adverse events in the control phase.

## Discussion

In this study, we have shown a significant effect of WBC on pain, impact of disease, and severity in a group of patients with FM and severe symptomatology and with mild undesired effects.

A study had already reported an improvement in quality of life with WBC in 50 patients with FM [22]. The WBC treatment protocol consisted of 15 sessions over a period of 3 weeks. Each session lasted 30 s with a temperature of − 60 °C followed by 3 min at − 140 °C. Improvement was demonstrated after treatment in pain, global health status, SF-36 and fatigue. The difference with our study relies on the temperatures reached, number of sessions, and design. The effect is demonstrated before after and compared to a group with no treatment.

The mechanisms of action of cryotherapy are not well understood. Since there is no proven inflammatory component in fibromyalgia, it has been postulated that cryotherapy through a reduction of oxidants levels may reduce muscular damage and accelerate recovery after normal physical activity. As a consequence pain and fatigue may substantially improve reducing symptomatology and improving physical function in these patients. Cryotherapy also relieves stress by the activation of neuroendocrine and metabolic functions and it is known that in patients with fibromyalgia stress is an important component [20, 28–30].

The crossover design allows all subjects receiving the treatment under study acting as their own control. This design is usually efficient, allowing smaller samples sizes due to reduced variability. However, we were affected by significant differences between the responses of the first and second period in the two outcome measures (VAS pain and total FIQ). That is, individuals did not return to baseline situation after the first treatment. The washout period was not long enough to ensure the disappearance of the effect of the treatment administered in the first period. We could not anticipate such a good outcome of WBC. In fact, based on the previous study by Bettoni et al. [22], we expected to need sessions on alternate days, therefore, a week seemed a proper duration of the washout period (three times the “half-life”). Future studies should contemplate lengthy washout periods in the experimental design to diminish the impact of carryover effects. Notwithstanding the barrier to include the cross-over period in the evaluation of the effect, differences before and after the intervention, even after the wash-out period and control phase, are significant in the group assigned to intervention in first place, suggesting a relevant residual effect of cryotherapy. Although a placebo effect can never be completely ruled out in a trial without a sham comparator, the observed effect is very pronounced and long-lasting, what merits further study. Also, the consequence of the carry-over effect is that the second period of the group assigned initially to cryotherapy cannot be added to the control arm, but this was not a major problem, as the differences were large enough to be detected even with a small number of patients. On the other hand, there was an unbalance at baseline in one of the endpoints; for this, the use of adjusted regression models made it possible to obtain results independent of the patient’s baseline situation. Another important limitation of our study was the open design. We could not design an appropriate sham therapy with the WBC cabin, as the temperature over which there is no therapeutic effect to use as placebo is unknown.

In summary, and taking into account the limitations of the study design, WBC during 3 weeks appears to produce a beneficial effect compared to no cold treatment in terms of pain and impact of disease in FM. The effect may last longer than a week after therapy, but will need to be demonstrated in future studies.

## Electronic supplementary material

Below is the link to the electronic supplementary material.

Supplementary material 1 (DOCX 17 KB)
