# Supplementary material for: The effect of cryotherapy on fibromyalgia: a randomised clinical trial carried out in a cryosauna cabin
Source: Rheumatol Int. 2018 Oct 23;38(12):2243–50. doi: 10.1007/s00296-018-4176-0 (PMC6223856; doi:10.1007/s00296-018-4176-0)
Supplement: Supplementary file 1 — Supplementary material 1 (DOCX 17 KB) [file 296_2018_4176_MOESM1_ESM.docx]

**Table 1.** Protocol schedule and assessments.

| Period | 1: Therapy / cryotherapy (+) | | | | | | | | | | | | | | | | | | | | | | WO | | | | | 2: Control / cryotherapy (-) | | | | | | | | | | | | | | | | | | | | | | | | | | | | | | | |
| --- | --- | --- | --- | --- | --- | --- | --- | --- | --- | --- | --- | --- | --- | --- | --- | --- | --- | --- | --- | --- | --- | --- | --- | --- | --- | --- | --- | --- | --- | --- | --- | --- | --- | --- | --- | --- | --- | --- | --- | --- | --- | --- | --- | --- | --- | --- | --- | --- | --- | --- | --- | --- | --- | --- | --- | --- | --- | --- | --- |
| Week | 1 | | | | | | | 2 | | | | | | | 3 | | | | | | | 4 | | | | | | 5 | | | | | | | | 6 | | | | | | | | 7 | | | | | | | | 8 | | | | | | | |
| Therapy | M | T | W | T | F | S | S | M | T | W | T | F | S | S | M | T | W | T | F | S | S | M | T | W | T | F | S | S | M | T | W | T | F | S | S | | M | T | W | T | F | S | S | | M | T | W | T | F | S | S | | M | T | W | T | F | S | S |
| FM criteria |  |  |  |  |  |  |  |  |  |  |  |  |  |  |  |  |  |  |  |  |  |  |  |  |  |  |  |  |  |  |  |  |  |  |  | |  |  |  |  |  |  |  | |  |  |  |  |  |  |  | |  |  |  |  |  |  |  |
| Pain VAS |  |  |  |  |  |  |  |  |  |  |  |  |  |  |  |  |  |  |  |  |  |  |  |  |  |  |  |  |  |  |  |  |  |  |  | |  |  |  |  |  |  |  | |  |  |  |  |  |  |  | |  |  |  |  |  |  |  |
| FIQ |  |  |  |  |  |  |  |  |  |  |  |  |  |  |  |  |  |  |  |  |  |  |  |  |  |  |  |  |  |  |  |  |  |  |  | |  |  |  |  |  |  |  | |  |  |  |  |  |  |  | |  |  |  |  |  |  |  |
| ICAF |  |  |  |  |  |  |  |  |  |  |  |  |  |  |  |  |  |  |  |  |  |  |  |  |  |  |  |  |  |  |  |  |  |  |  | |  |  |  |  |  |  |  | |  |  |  |  |  |  |  | |  |  |  |  |  |  |  |
| SF-36 |  |  |  |  |  |  |  |  |  |  |  |  |  |  |  |  |  |  |  |  |  |  |  |  |  |  |  |  |  |  |  |  |  |  |  | |  |  |  |  |  |  |  | |  |  |  |  |  |  |  | |  |  |  |  |  |  |  |
| Adverse events |  |  |  |  |  |  |  |  |  |  |  |  |  |  |  |  |  |  |  |  |  |  |  |  |  |  |  |  |  |  |  |  |  |  |  | |  |  |  |  |  |  |  | |  |  |  |  |  |  |  | |  |  |  |  |  |  |  |
| Visits | V  1 |  |  |  |  |  |  |  |  | V  2 |  |  |  |  |  |  |  |  |  |  |  | V  3 |  |  |  |  |  |  | V  4 |  |  |  |  |  |  | |  |  | V  5 |  |  |  |  | |  |  |  |  |  |  |  | | V  6 |  |  |  |  |  |  |

Baseline visits are V1 and V4. Evaluation of results: V3 and V6.

WO = washout period.
